# Validation of digital biomarkers

**DOI:** 10.1002/alz.083419

**Published:** 2025-01-03

**Authors:** Rhoda Au, Lampros Kourtis

**Affiliations:** ^1^ Department of Epidemiology, Boston University School of Public Health, Boston, MA USA; ^2^ Department of Anatomy and Neurobiology, Neurology and Medicine, Framingham Heart Study, BU Alzheimer’s Disease Research Center, Boston University Chobanian & Avedisian School of Medicine, Boston, MA USA; ^3^ Davos Alzheimer’s Collaborative, Philadelphia, PA USA; ^4^ Gates Ventures, Seattle, WA USA; ^5^ Clinical and Translational Science Institute, Tufts University, Boston, MA USA

## Abstract

**Background:**

Interest in use of digital technology to advance AD/ADRD research has been growing exponentially over the last few years. This acceleration is fueled in part by growing awareness that both well used research methods as well as newer biomarker approaches are 1) inadequate for clinical symptom detection in the earliest stages of an insidious onset disease and 2) have resulted in inaccurate as well as biased data that is generating treatment and prevention solutions that are insufficiently relevant to some and potentially not relevant to many.

**Methods:**

Sensors embedded in mobile devices such as smartphones and wearables deliver a high penetration, low‐cost solution for overcoming previous limitations of early detection sensitivity and limited representative reach. The advances in AD biomarker development has spilled over into the digital realm, leading to the misconception and misclassification of many digital measures as digital biomarkers.

**Results:**

Re‐examination of what is and is not a digital biomarker today and rethinking what a digital biomarker could be tomorrow is warranted. The validation pathways for digital biomarkers are twofold: 1) the path of least resistance, which creates digital versions of what is currently already being done and 2) the path of greatest resistance, where the definition of such is still unknown, including to the FDA.

**Conclusion:**

The impact of digital biomarkers today is still rather modest because research and clinical care remain relatively risk adverse. Re‐imagination not bounded by precedent will be needed to unleash the full potential of what digital biomarkers could and should be, which include well beyond what can be conceived of currently.

